# Dr. Oliver Wendell Holmes

**Published:** 1855-09

**Authors:** 


### DR. OLIVER WENDELL HOLMES.

OUR citizens have just been favored with a visit by this eminent physician, scholar and poet. He came to Louisville at the invitation of the "Young Men's Christian Association" to deliver a series of lectures on the Modern Poets of England. We had the pleasure of listening to several of his lectures, which abounded in wit, humor, poetry, and fine philosophical analysis. When these lectures are published we have no hesitation in saying that they will add to the reputation of Dr. Holmes as a writer, high as he now deservedly ranks among the literary men of the day. The "magnetic stone of genius" has assuredly fallen at his feet, and whether he writes prose or verse the divine gift is at once seen and felt by his readers. The Association of young men who brought Dr. Holmes to the city is entitled to the thanks of the community. It gives us a better opinion of our country to listen to its eloquent orators and poets, and draws closer the bonds between the distant portions of it to bring its citizens together in the interchange of hospitality and friendly offices.

---

AT the late annual dinner of the Massachusetts Medical Society, held at Springfield, on the 27th of June, this eminent poet-physician made, in reply to a toast, the following well-timed remarks:

"We have need of every humanizing influence, of every form of culture, to lend grace and dignity to our position. We are assailed as we never were before. Journalists are running their setons through our necks, and keeping every sore spot open with their daily epispastics, sprinkled with the cantharides of their "machine-spread blistering plasters." Legislatures are debating whether to build costly hot-houses to force a growth of mushrooms—no, of toadstools—into existence among the roots of the bread-tree, which so many centuries have nourished with the life-blood of true-hearted and sound-minded men. The so-called 'Reformer,' whose incisors, like those of the rodentia, grow into his own flesh so soon as he finds nothing hard to gnaw upon, is mumbling at our unguarded extremities.

"And in the meantime, we must not disguise it from ourselves, that causes are at work, within as from without, that may well occasion grave thought on the part of those who are anxious that the profession should maintain its high standing. Medical practice is breaking up into *specialities*—that make men skillful and narrow-minded. The Egyptians had their specialists for each organ, and the indications are unequivocal of a return towards the state of primitive Egyptian—culture. I do not say that it is not better for mankind that our art should be thus subdivided, but it is full of danger to the social and intellectual standing of the profession. It is now six years, I think, since, in a written introductory lecture, I distinctly pointed out to my class the course which medical practice was shaping for itself. Every succeeding year has confirmed what I then said. The path to *glory* (?) in medicine now leads through the—mucous membrane. Each of these slippery highways is invaded by its exclusive manipulator. One man passes his days in cauterizing throats and swabbing out bronchial passages. Another explores the narrow track from the fossa navicularis to the verumontanum, and there lives and moves and has his being. Another mainly studies science through the valves of a speculum. Another selects a still humbler sphere and holds divided empire with the ascaris!

"This subdivision of labor ends in most cases in the production of at least one cheap and perfect article, namely, a piece of human machinery capable of doing one thing well, and good for little else in many cases, stiffened, cramped, anchylosed in all the intellectual joints that give freedom and power to manhood. *Now there is nothing that can rescue the profession, engaged in such a track, from degradation to the rank of the mere mechanical arts, but a high standard of education and accomplishments.*

"There is something else besides the skillful hand, and the brain filled with the minute detail of some speciality, necessary to bring the physician into healthy and dignified relations with the intelligence of a wide-awake community like ours. We want men whom we can point out to the world and say: Look at them; and not men to place in a corner and say to our patients: Go, let them feel of you! Depend upon it, if our general culture does not, in some degree, keep pace with that of our enlightened clients, we shall become 'servants,' as old Wiseman used to call his own professional assistants; valets of the sick man, and fit companions for the flunkies that comb and brush him in health. And so, at last, the small specialist, pedicure or anicure, will do his small service, and be shown out the back door, as their predecessors, the Egyptian craftsmen, when they had performed their office of making the necessary incisions for the embalmer, were driven from the house with their fee in their pockets, and such convenient missiles flying about their heads as the friends could lay hands on.

"I have pointed out a danger, but not without suggesting a remedy. In a word, to keep our profession what it has been, worthy of such an eulogy as Pope bestowed on it, fit calling for such men as Haller, as Arbuthnot, as Abercrombie, as our professional brother* now languishing in illness, which he charms at once with philosophy and song, we must give it every attraction which large aims and liberal culture can add to its inherent usefulness, so that we may call to it that noble class of minds out of which alone the time heroes of our divine science can be moulded."

He concluded his speech with the following beautiful poem:

As the mountain of New England,
In the far off Northern sky,
Looks down upon the river
That wanders murmuring by;
So from her cloud-capped turret,
Let solemn art look down
On fashion's lying whisper,
And folly's idle frown.

As the river of New England,
That is flowing at our side,
No rock can stay from running
To meet the salt sea-tide;
So roll our art's deep current,
By many a graceful shore,
Till life has reached the ocean
That pain shall vex no more!

As the bright star of evening
Plays on the mountain's crest,
Glancing from every streamlet
That sparkles on its breast;
So let the heavenly splendors
Of truth's white star be found,
Still shining fairest in us
While all is dark around!

*Virginia Med. and Sur. Journal.*

* Elisha Bartlett, M. D., since deceased.

